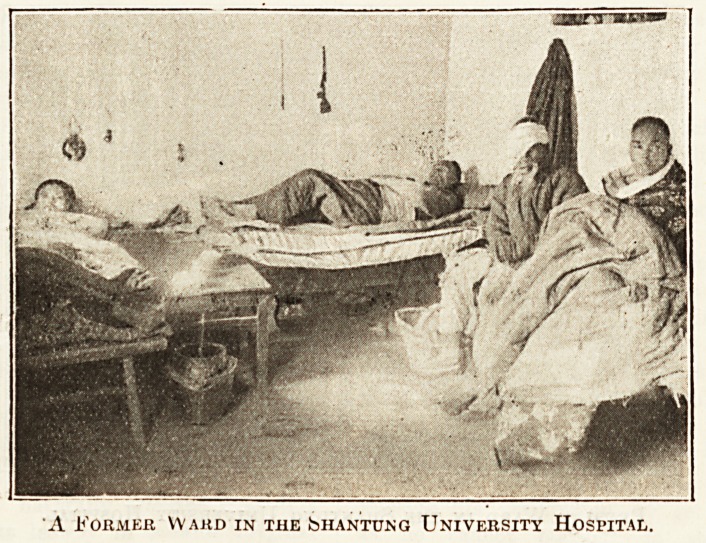# Two Modern Schools and Hospitals

**Published:** 1921-03-05

**Authors:** 


### MEDICAL EDUCATION IN CHINA.

### Two Modern Schools and Hospitals.

Two hundred years ago medical science in China was not far behind that of any other country in the world. Since that time, in the absence of medical colleges and hospitals, or of Government regulations safeguarding the practice of medicine, scientific progress has been nil. It is only of recent years that the Chinese Government has taken any steps to establish a system of medical education. Meanwhile, however, the medical missions throughout China have been at work; they have set up standards of Christian service, have gradually opened up mission hospitals in various places, and have provided for Chinese students of good education the opportunity to devote themselves to the study of medicine and to qualify for its practice according to modern standards.

### The Peking Union Medical College.

Two of the most important medical colleges in China are the Peking Union Medical College and the Shantung University Hospital and Medical School at Tsinan. A more dramatic story of growth than that of the former is difficult to find. After the Boxer outbreak a little hospital in Peking, owned by the London Missionary Society, had been shattered. A young doctor, who had been sent up into Mongolia following the death of the pioneer Dr. Gilmour, was brought back by his Society to Peking, and instructed to rebuild the medical work. The old Empress was on the throne. As a "foreign devil," he was refused access to the forbidden city. Everything seemed against him. Yet, by curing the Prime Minister of the old Empress of a dangerous disease, he not only secured audience of the Empress herself, but demonstrated to her the stupendous possibilities of good for her country that lay in teaching the Chinese doctors, surgeons, and nurses to minister to the medical needs of their own people. She made large contributions to the work, which were followed by others from Viceroys. A great expansion became possible. The doctors of a number of different missionary societies—Anglican and Free Church—operating in China got together and constructed a plan for a Union Medical College which should be at once international (that is, including America and Britain) and inter-denominational.

The plan was brought to fruition, and Dr. Cochrane, the young doctor of whom we have spoken, became first Principal. The Government made it a recognised college that could grant degrees in medicine, and a very high standard of teaching and training was reached.

In the spring of 1914 the Rockefeller Foundation sent the China Medical Commission "to inquire into the condition of medical education, hospitals, and public health in China." In their recommendations, the Commission advised that the first medical educational work organised should be in the city of Peking, and that it be in connection with the Union Medical College if suitable arrangements could be made. Following this, the China Medical Board of the Rockefeller Foundation was created. The first important act of the China Medical Board was the acquisition of the property of the Union Medical College in Peking. The terms of the transfer provided, among other things, that the work of the College should be conducted by a Board of Trustees, which should consist of thirteen members, one to be appointed by each of the six missionary organisations previously maintaining the College, and seven by the China Medical Board. On July 1, 1915, the China Medical Board assumed the full support of the College. Early in 1916 a provisional Charter was secured from the Regents of the University of the State of New York, and in accordance with this Charter the Trustees adopted by-laws and leased from the China Medical Board the property of the Medical School in Peking.

Present Ward in the Shantung University Hospital.

The requirements for admission are based on those of the New York State of Regents of the Association of Medical Colleges and of the Chinese Ministry of Education for Medical Schools.

The minimum requirements for admission include graduation from an approved middle school, with at least 108 credit hours of college work in addition, two hours of laboratory being counted as one hour of class-room work, the work including English, Chinese, French, mathematics, biology, physics, and chemistry. Although the general level is as high as that for an American or British course, special additional emphasis is given to parasitology, general and advanced. The work of the reorganised College (which was opened in October 1919) is divided between two schools: the medical, with a four-years' course in medicine and one of special work in laboratories or hospital; and the pre-medical (opened in September 1917), which offers a three-years' course preparatory to admission to the medical school. The English language is the medium of instruction, but with the view of the ultimate probable adoption of Chinese certain courses to insure progress in the students' own language are offered and required.

The College, with its pre-medical and medical schools, is now (during this last year) for the first time open to women students, with the same requirements for admission and graduation as in the case of men. There are separate hostels being provided for the women, and a training school for nurses is attached. An interesting feature is a small private pavilion for patients to secure the professional services of the staff without entering the public wards.

**Medical Education in China**—(*continued*).

The nurse-training school, under the direction of Miss Anna Dryden Wolf, M.A., R.N., is on modern lines, and graduation from a middle school is required for admission.

---

### The Shantung University Hospital and Medical School, Tsinan.

This is an amalgamation of three medical colleges—those at Nanking, Hankow, and Tsinan—and is the outcome of the recommendation of the Council of Medical Education of the China Medical Missionary Association in order to ensure the establishment of a high-grade medical college teaching in the Chinese language. To this amalgamated school the three junior classes of the Peking Union Medical College have been transferred to complete their medical education at the request of the Rockefeller Foundation, which, in return, made a grant of £37,500 towards the cost of additional buildings and equipment, and of a larger staff—a grant which has since been extended. Dr. Harold Balme, the Dean of the medical school at Tsinan, considers this amalgamation one of the most remarkable instances of missionary co-operation in China. The co-operating societies are the Baptist Missionary Society, the Society for the Propagation of the Gospel, the London and the Wesleyan Missionary Societies (all British), the Canadian Presbyterian Mission, and the American Presbyterian Missions (North and South), the Norwegian Lutheran Mission, and the Grinnell-in-China Movement (American). The English Presbyterian Mission also makes a small annual grant.

A Former Ward in the Shantung University Hospital.

Tsinan is the capital of the Province of Shantung—known as the Sacred Province by reason of it having been the home and burial-place of Confucius and Mencius—and has a population of 350,000. The students in the school of medicine number ninety-eight, and in the pre-medical department forty-six; they come from fourteen provinces and represent eighteen denominations. They are required to spend at least two years in pre-medical studies, modelled on those in use in science colleges and medical schools in Great Britain and North America, before entering on the five years' medical curriculum. The last three years of the latter course are devoted to clinical subjects, and in the fifth year class work is reduced to a minimum, the students' time being chiefly concentrated on clinical investigations both in the wards and in the out-patient department. Particular attention is paid in the curriculum to the teaching of physiological chemistry, with the view of inducing students in future years to undertake original research into such vital subjects as Chinese dietetics and the comparison of Chinese physiological processes with those investigated in Western countries. Funds are needed to erect a new laboratory block so as to extend the accommodation for the pathology and bacteriology departments, and to provide accommodation for post-graduate study. In connection with this block it is hoped to organise a department of preventive medicine, the study of which is now looked upon in China as of the highest importance. The work already done in this connection has borne good fruit, for the pneumonic plague of 1918 was stamped out in Tsinan in one month, with only sixteen deaths, and a similar good result was obtained during the cholera epidemic of 1919.

The original buildings of the medical school were erected in 1909, and consisted of a college block, students' dormitories, three residences, and a small hospital. In 1914 a new hospital and out-patient department were erected, and in 1917 the main school building was entirely rebuilt and added to. Our illustration of a former hospital ward was taken as recently as 1913, and speaks for itself. The present hospital contains 110 beds, is provided throughout with central heating and electric lighting, and is equipped and furnished on modern lines; patients are admitted and treated as in Western hospitals. This accommodation is, however, inadequate. As soon as the necessary funds are forthcoming it is hoped to erect a separate hospital for women and children and to enlarge the present building for male patients.

#### The Nurse Training-School.

With the rebuilding of the hospital in 1914 and the advent of the Chinese trained medical man, the institution of modern nursing became possible. In 1914 the first class of Chinese nurses, all graduates from mission schools, was received into the University Hospital for training on lines corresponding to those of nurse training-schools in this country. Miss Margaret F. Logan is the superintendent of nursing, and she is assisted by five trained nurses. The probationers number about forty. Nurses as they graduate assist in the supervision of ward work, both at Tsinan and elsewhere. The course of instruction is four years.

China has its Nurses' Association, which grants a diploma in nursing to all graduates of registered training-schools who pass the standardised examinations which such schools must adopt. Over fifty training-schools have already been registered. In the matter of the standardisation of nurses' education and examination, as well as in the requirement of pre-medical training for medical students, China claims to be ahead of this country.

The School of Medicine and University Hospital, Tsinan, receives an increasing proportion of its income each year from the fees of students and patients and the contributions of Chinese officials and others, but it still depends to some extent on contributions from Great Britain and America. Its more urgent needs at present amount to upwards of £130,000, and include the provision of a tuberculosis sanatorium, extension of the out-patient department, a hospital for private patients, and a home for Chinese nurses, in addition to the buildings mentioned earlier in this article.

## Figures and Tables

**Figure f1:**
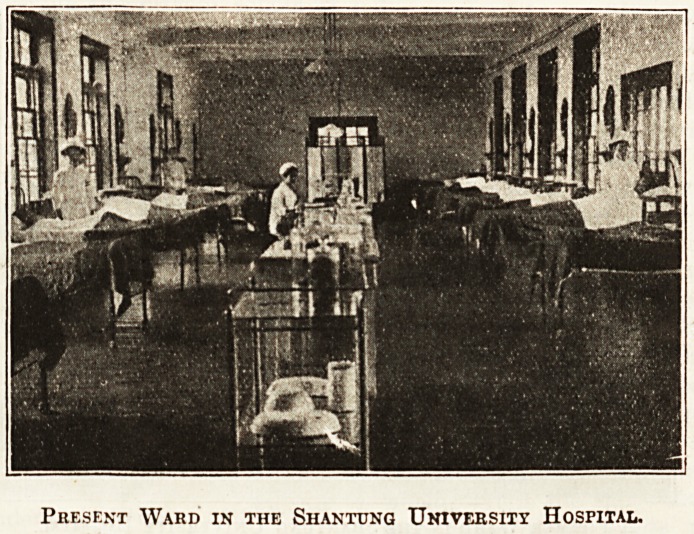


**Figure f2:**